# The association between telomere length and cancer risk in population studies

**DOI:** 10.1038/srep22243

**Published:** 2016-02-26

**Authors:** Xun Zhu, Wei Han, Wenjie Xue, Yuxia Zou, Cuiwei Xie, Jiangbo Du, Guangfu Jin

**Affiliations:** 1 Department of Epidemiology and Biostatistics, the Collaborative Innovation Center for Cancer Personalized Medicine, School of Public Health, Nanjing Medical University, Nanjing 211166, China; 2 Xuzhou Center for Disease Control and Prevention, Xuzhou 221003, China

## Abstract

Telomeres are crucial in the maintenance of chromosome integrity and genomic stability. A series of epidemiological studies have examined the association between telomere length and the risk of cancers, but the findings remain conflicting. We performed literature review and meta-analysis to demonstrate the relationship between telomere length and cancer risk. A total of 23,379 cases and 68,792 controls from 51 publications with 62 population studies were included in this meta-analysis to assess the association between overall cancer or cancer-specific risk and telomere length. General association and dose-response relationship were evaluated based on two and three groups, respectively. The estimates of association were evaluated with odds ratios and 95% confidence intervals by the random-effects or fixed-effects model based on heterogeneity test. We observed a non-significant association between short telomeres and overall risk of cancer. Convincing evidence was observed for the association of short telomeres with an increased risk of gastrointestinal tumor and head and neck cancer. Significant dose-response associations were also observed for gastrointestinal tumor and head and neck cancer. Our findings indicate that telomeres may play diverse roles in different cancers, and short telomeres may be risk factors for the tumors of digestive system.

Telomeres consist of several thousand DNA repeats of *TTAGGG* in association with a protein complex at the ends of chromosomes in eukaryotic cells. Telomeres maintain chromosome integrity and genomic stability through prohibiting nucleolytic degradation, chromosomal end-to-end fusion and irregular recombination^1^,^2^. In humans, the average telomere length ranges from 10 to 15 kb^3^, and telomeric DNA shortens during each cell replication at a rate of 50–200 bp^4^. In general, a critically short telomere length can trigger cell to enter replicative senescence with a result of cell death^5^,^6^; alternatively, cells continue to divide if death does not occur, which results in genomic instability and chromosomal abnormality. Therefore, telomere length acts as a mitotic clock for eukaryotic cells, and potentially represents the number of cell replications undertaken by each cell during its lifespan^7^.

Telomeres are strongly correlated between tissues, and the rates of telomere shortening are also similar^8^. Telomere length in leukocytes is considered as useful surrogate for the other tissues. Numerous epidemiological studies have focused on analyzing the telomere length in peripheral blood cells in relation to various diseases, including multiple cancers. However, the reported findings are conflicting. In 2011, two meta-analysis^9^,^10^ pooling more than 20 studies reported that the short telomeres were associated with increased cancer risk. They also found particularly strong evidence for bladder, esophageal, gastric, and renal cancers, but the study numbers were limited for each cancer type. Afterwards, emerging studies with relatively large sample size investigated the association between telomere length and cancer risk. However, the findings are still conflicting other than conclusive, particularly for different cancer types. Nevertheless, more and larger studies may allow for stronger statistical power for meta-analysis, especially for single cancer type. Herein, we carried out a systematic review and meta-analysis on 56 relevant literatures^11^,^12^,^13^,^14^,^15^,^16^,^17^,^18^,^19^,^20^,^21^,^22^,^23^,^24^,^25^,^26^,^27^,^28^,^29^,^30^,^31^,^32^,^33^,^34^,^35^,^36^,^37^,^38^,^39^,^40^,^41^,^42^,^43^,^44^,^45^,^46^,^47^,^48^,^49^,^50^,^51^,^52^,^53^,^54^,^55^,^56^,^57^,^58^,^59^,^60^,^61^,^62^,^63^,^64^,^65^,^66^ to estimate the overall cancer risk or cancer-specific risk associated with telomere length and to evaluate potential between-study heterogeneity of these studies.

## Materials and Methods

### Search strategy and selection criteria

We conducted a literature review using PubMed to identify reports on an association between telomere length and cancer risk through to May 31, 2015. The search terms were “telomere length”, “cancer” or “carcinoma”, and “risk”. We limited the publication language to English. The criteria included: 1) a case–control or cohort study design assessing the relationship between telomere length and cancer risk; 2) sufficient information for estimating odds ratios (ORs) and their 95% confidence intervals (CIs); 3) without overlap between studies in terms of study subjects.

### Data extraction

The following data was extracted from each publication: the first author, year of publication, country, ethnicity, cancer type, the number of cases and controls grouped by median, tertiles, quartiles or quintiles of relative telomere length (T/S ratio), study design, DNA source, and method for telomere length measurement. Data was extracted separately for studies including subjects from different ethnicities, multiple cancer types or independent populations if possible. Because controls were shared for multiple cancers in two publications^11^,^47^, each publication was divided into multiple studies in the cancer-specific analysis but treated as one study by pooling all cancer cases together as compared with shared controls. When multiple publications had the same or overlapping subjects, only the largest or latest studies were included.

### Quantitative data synthesis

To simplify the analysis, we firstly collected the number of cases and controls from two groups (short and long) divided by the median telomere length for each study to evaluate the association. Because some studies reported data in three or five groups based on tertile or quintile value, we treated the groups of “Q1 and Q2” or “Q1, Q2 and Q3” as the short groups, respectively, and the other groups as the long groups. In the sensitivity analysis, we also performed analysis by dividing the subjects into three groups (short, medium and long). We combined Q2 and Q3 groups as the medium group for studies including four groups (Q1, Q2, Q3, Q4), and combined Q1 and Q2 groups as the short group, and Q4 and Q5 groups as the long group for studies including five groups (Q1, Q2, Q3, Q4, Q5). Two publications^29^,^47^ providing the numbers of two groups only were excluded in this analysis. The association between the telomere length and cancer risk was examined by ORs and 95% CIs with the group of long telomeres as the reference. We performed cancer-specific analysis by cancer type and the cancer types reported in less than 3 studies were merged into the “other types of cancer” group. Gastrointestinal tumor included those diagnosed in the stomach, esophagus, colon or rectum. Cancers arising from the bladder, kidney and prostate sites were considered tumors of the urogenital system. We also performed analysis by study type (retrospective and prospective) and ethnicity (Caucasian, Asian or African American).

The χ^2^-based Q test was performed to evaluate between-study heterogeneity and considered significant if *P* < 0.10^67^. Heterogeneity was also quantified with the *I*^2^ statistic that indicates what proportion of the total variation across studies is beyond chance. The value of 0% indicates no observed heterogeneity and larger values show increasing heterogeneity^68^. The fixed-effects model and the random-effects model were used to pool the data from different studies based on the Mantel-Haenszel method and the DerSimonian and Laird method, respectively^69^.When the *P* value of the heterogeneity test was ≥0.10, the fixed-effects model was used, which assumes the homogeneity of effect size across all studies. Elsewise the random-effects model was more appropriate, which tends to provide wider confidence intervals, when the results of the constituent studies differ among themselves. Potential publication bias was evaluated with funnel plots of effect sizes versus standard errors. Begg’s test was used to examine the significance of asymmetry at a significance value of 0.10. All analysis was conducted by using Review Manage (v.5.3) and R3.0.1.

## Results

### Characteristics of Studies

**Figure 1:**
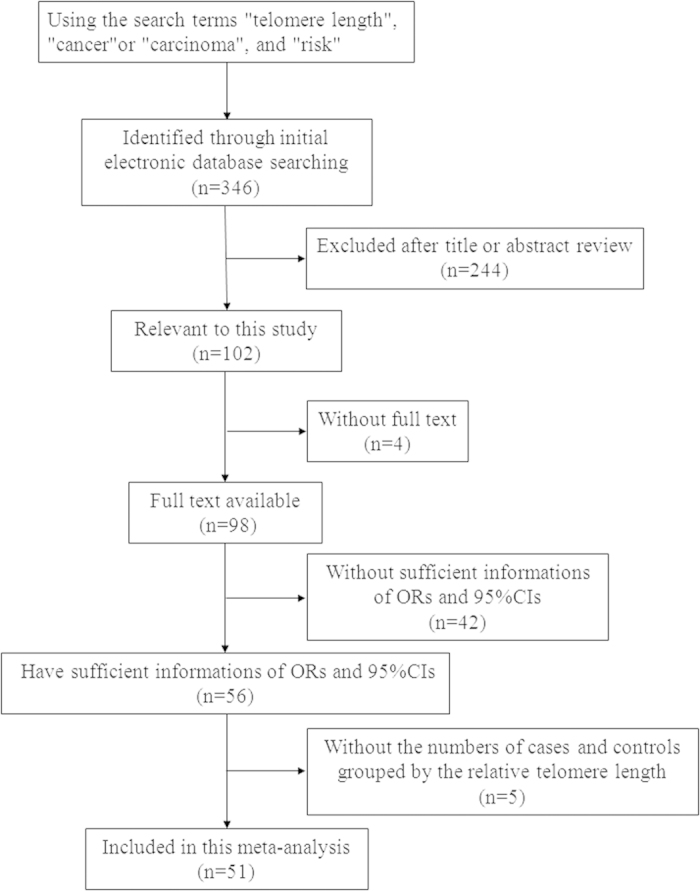
Flow chart for the process of selecting the final 51 publications.

A total of 56 publications were identified with an evaluation of the association between telomere length and cancer risk (Fig. 1). Five reports were excluded because they did not provide the numbers of cases and controls grouped by the relative telomere length^62^,^63^,^64^,^65^,^66^. The remaining 51 publications contained 62 studies (Xifeng Wu’s study^16^ had datasets of four different cancers; Gabriella M. Anic’s^11^ and Jiali Han’s^13^ studies had three datasets of different cancers and Geyu Liang’s^14^, Beatriz Sanchez-Espiridion’s^43^, and Yang Zhang’s^47^ studies had two datasets of different cancers, and Jonathan N. Hofmann^21^ had two datasets of independent populations. We summarized the general information of these 62 studies in Table 1. There were 10 studies for skin cancer^11^,^12^,^13^,^14^,^15^ and tumors of urogenital system^16^,^17^,^18^,^19^,^20^,^21^,^22^,^23^, 9 for gastrointestinal tumor^24^,^25^,^26^,^27^,^28^,^29^,^30^,^31^,^32^, 8 for breast cancer^33^,^34^,^35^,^36^,^37^,^38^,^39^,^40^ and lung cancer^16^,^41^,^42^,^43^,^44^,^45^,^46^, 4 for head and neck cancer^16^,^47^,^48^, 3 for lymphoma^49^,^50^,^51^, and 10 for the other types of cancer with each type less than 3 studies^52^,^53^,^54^,^55^,^56^,^57^,^58^,^59^,^60^,^61^ (Table S1). Most of studies (n = 51) recruited subjects from populations of Caucasian descent, 10 studies of Asian descent, and one study of African American descent. The quantitative PCR was used to measure the relative telomere length (T/S ratio) in 55 studies, whereas fluorescence *in situ* hybridization (FISH)-based assays were used in 7 studies^16^,^39^,^45^. Additionally, blood cells were main DNA source except one study based on circulating cell-free serum DNA^54^.

### Quantitative Synthesis

We obtained the telomere length data from 51 publications consisting of 23,379 cases and 68,792 controls. When pooling all eligible studies into the meta-analysis, we found a non-significant association between short telomeres and an increased risk of overall cancer risk (OR = 1.10, 95% CI: 0.98–1.23, Table 2). The directions of association were consistent among three populations from different descents (ORs = 1.08, 1.15 and 1.22 for Caucasian, Asian and African American, respectively, Table 2). The results of analysis for subgroups of different ethnicities have been shown in Table S2. However, the association was disappeared in prospective studies (OR = 1.02, 95% CI: 0.87–1.19). Moreover, we also excluded three prospective studies^28^,^59^,^61^ from the meta-analysis and found the similar results for overall cancer risk (OR = 1.07, 95% CI: 0.95–1.21).

**Figure 2:**
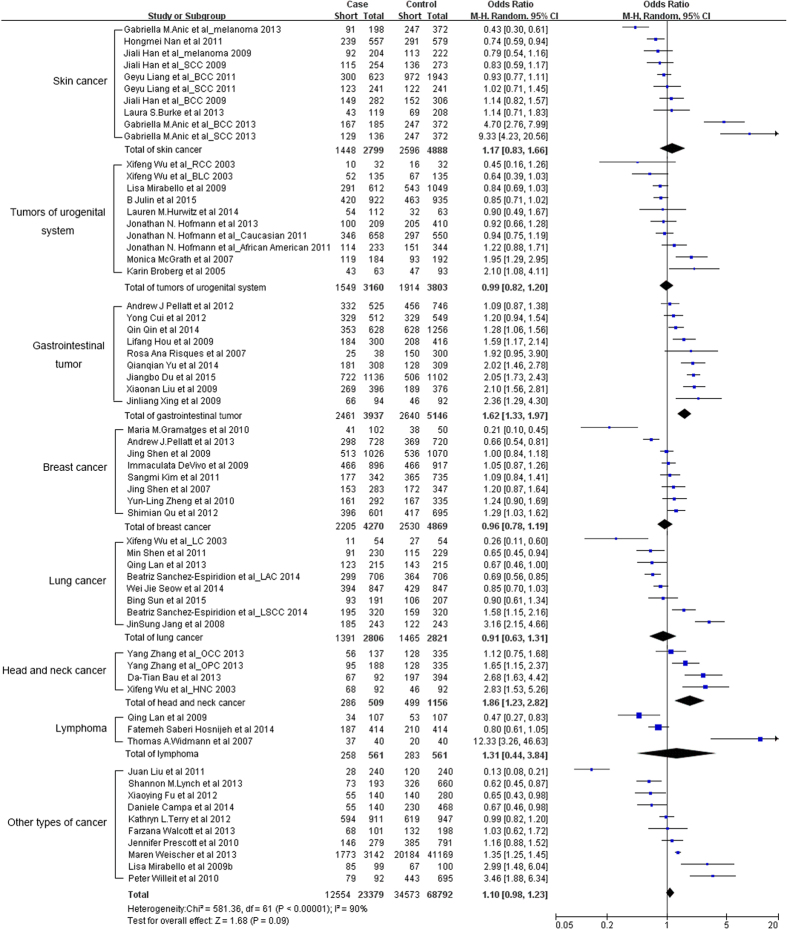
ORs and 95% CIs for cancer risk associated with telomere length (short vs. long).

Considering that heterogeneity is extensively occurred across cancer types, we then performed cancer-specific analysis (Fig. 2). Short telomeres were significantly associated with increased risks of gastrointestinal tumor (OR = 1.62, 95% CI: 1.33–1.97) and head and neck cancer (OR = 1.86, 95% CI: 1.23–2.82). Of interest, in prospective studies rather than retrospective studies, short telomeres were associated with a decreased risk of lung cancer (OR = 0.78, 95% CI: 0.67–0.91). There was no obvious evidence supporting the association for the other cancer types (Table 2).

To evaluate the robustness of pooling results based on dichotomized telomere length, we further divided the cases and controls into three respective groups for each study, and tested the dose-response relationship between telomere length and cancer risk by pooling the studies together. We observed a significant increased risk of overall cancer for short telomeres with a trend OR (95% CI) of 1.09 (1.01–1.19) (Table 3). In cancer-specific analysis, dose-response effects of telomere length were also detected on gastrointestinal tumor (OR = 1.29, 95% CI: 1.08–1.54), and head and neck cancer (OR = 2.30, 95% CI: 1.74–3.02), which were consistent with the above results based on dichotomized telomere length (Table 3).

### Heterogeneity analyses

Substantial heterogeneity was observed among all studies for the association between telomere length and cancer risk (*P* < 0.001, *I*^ 2^ = 90%, Fig. 2). We then evaluated the potential source of heterogeneity and found significant effect difference between subgroups for cancer type (*P* < 0.001), study design (*P* = 0.008), and ethnicity (*P* < 0.001).

### Publication bias

**Figure 3:**
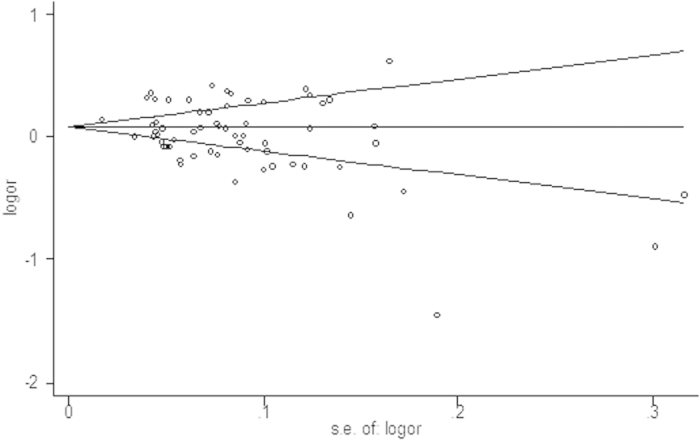
Funnel plot analysis to detect publication bias.

The shape of the funnel plot seemed symmetrical (Fig. 3), and the Begg’s test did not show a significant publication bias in the current meta-analysis (*P* = 0.142). These indicated that bias from publications might not have a significant influence on the results of our meta-analysis on the association between telomere length and cancer risk.

## Discussion

In this study, we performed the largest and most comprehensive literature review and meta-analysis on the association of telomere length and cancer risk, including a total of 23,379 cancer cases and 68,792 controls from 51 independent publications. We did not find significant association between telomere length and overall risk of cancers, but showed a robust association with gastrointestinal tumor and head and neck cancer. In addition, we also observed promising association of short telomeres with a decreased lung cancer risk in the prospective studies. Furthermore, dose-response relationships provided further evidence for the associations with gastrointestinal tumor, and head and neck cancer.

Telomeres are specialized structures that protect chromosome ends and participate in a number of processes of a great cellular relevance^70^, which makes the telomere crucial in cellular senescence and carcinogenesis^71^. Progressive telomere shortening occurs with each cell division up to a point termed “replicative senescence” in most human somatic cells^72^. Basic biology studies have established that telomere shortening is a fundamental feature of dividing cells and directly related to the age of the cell lineage, and that telomere crisis in the present of defective cell-cycle control can lead to chromosomal instability and a malignant phenotype^73^. The dysfunctional telomeres will result in chromosomal fusions, continuous “breakage-fusion-bridge” cycles, derived chromosome imbalances, gene amplifications, and ultimately the generation of complex non-reciprocal translocations, a hallmark feature of adult solid tumors and genomic instability in general^74^. At the population level, the high incidence of cancer has prompted that shortening of telomeres promotes tumor development and several studies have found that patients with shorter telomeres in peripheral blood cells have a higher risk of developing carcinomas^75^. In this meta-analysis, although we found there is no significant association between telomere length and overall risk of cancers, but we demonstrated a significant association with gastrointestinal tumor and head and neck cancer, supporting the hypothesis that excessive telomere shortening may play an important role in accelerating tumor onset and progression. Gastrointestinal tumor and head and neck cancer is kind of epithelial malignancies in digestive system. The majority of epithelial malignancies appear to develop from morphologically defined precursor lesions termed intraepithelial neoplasia^76^. Telomere length in more than 90% intraepithelial neoplasia is dramatically shortened^77^. In addition, telomeres of gastrointestinal tumor may exhibit an intensified rate of shortening that is greatly accelerated as compared to the normal tissue of origin^78^.

However, our results revealed heterogeneous association results between different cancer types. Short telomeres were convincingly associated with increased risk of gastrointestinal tumor and head and neck cancer, which, however, was not observed in other types of cancer. Of note, a significant but inverse association was shown for lung cancer in prospective studies. These inconsistent results across cancer types may reflect different carcinogenic mechanisms conferred by specific telomeres in specific cancer types. For example, several studies^11^,^12^ found a higher risk for melanoma among individuals with longer telomeres, this may suggest that shorter telomere lengths protect against the malignant transformation of cells within melanocytic nevi by limiting proliferative capacity and triggering the entry to senescence stage. To the contrary, longer telomeres were found to be protective for basal cell carcinoma (BCC) and squamous cell carcinoma (SCC) with the reason of that UV exposure may be more likely to induce genomic abnormalities in cells with shorter telomeres. In addition, Sanchez-Espiridion *et al.*^43^ found that patients with lung adenocarcinoma had longer telomeres than controls, whereas patients with lung squamous cell carcinoma had shorter telomeres compared with controls. These findings suggest that telomere length may affect cancer risk in a histologic manner, further highlighting the distinct roles of telomere in cancer development.

In addition to the cancer-specific associations, telomere length may also involve in cancer risk in a complex manner rather than a simple linear relationship. Cui *et al.*^25^ reported a U-shaped association between telomere length in peripheral blood cells and colorectal cancer (CRC) risk, and they found that both very short and very long telomeres are risk factors for colorectal cancer. Recently, we also reported a non-linear relationship between telomeres and gastric cancer risk^28^. Similar results were also reported in pancreatic cancer^64^, breast cancer^40^ and glioma^66^. These observations are also biologically plausible because telomeres may act as a double-edged sword in the development of cancer. Telomere shortening can generally lead to chromosomal instability and finally initiate the process of carcinogenesis^16^. However, long telomeres may allow for more cell divisions and increase the chance of acquiring abnormalities for cancer development^79^. However, due to lack of original data, we cannot evaluate this phenomenon in this study. Further studies are warranted to carefully test these findings.

There are some limitations in this meta-analysis. Firstly, some factors can affect the length of telomeres, such as age, gender, and tobacco smoking, and oxidative stress^80^,^81^. The results of this meta-analysis were based on unadjusted estimates, because odds ratios (ORs) derived from different studies were not adjusted by the same potential confounders or only the number of cases and controls was provided without the detailed information of other variables. Secondly, we performed analysis by dividing the subjects into two or three groups simply due to lack of original data of relative telomere length, which may decrease the power to evaluate the relationship of telomere length and overall risk of cancers. In the main analysis of this study, we treated the groups of “Q1 and Q2” (for three groups) or “Q1, Q2 and Q3” (for five groups) as the short groups, and the other groups as the long groups. To address the stability of the results, we also treated the groups of “Q1” (for studies with three groups) or “Q1, and Q2” (for studies with five groups) as the short groups and the other groups as the long groups, and found that the results were similar (OR = 1.08, 95% CI: 0.96–1.22 for overall cancer risk).

In summary, our meta-analysis provided strong evidence for the association between short telomeres and increased risk of gastrointestinal tumor and head and neck cancer. In addition, the short telomeres also increased, although not significantly, the risk of overall cancer in the analysis of dichotomized variable, this association may be influenced by the study numbers of different tumors because the effects are different between tumors. However, larger, well-designed prospective studies are needed to validate these findings, which may help to uncover the potential mechanisms of telomere dysfunction in cancer development.

## Additional Information

**How to cite this article**: Zhu, X. *et al.* The association between telomere length and cancer risk in population studies. *Sci. Rep.* **6**, 22243; doi: 10.1038/srep22243 (2016).

## Supplementary Material

Supplementary Information

## Figures and Tables

**Table 1:** Information summary of 51 eligible studies included in this meta-analysis.

Author [reference]	Country	Year	Cancer type	Ethnicity	No. of case/control	Study type	Control source	DNA source	Measurement methods
Gabriella M. Anic *et al.*_melonoma^11^ ^a^	USA	2013	melanoma	Caucasian	198/372	retrospective	hospital-based	leukocyte	quantitative PCR
Gabriella M. Anic *et al.*_BCC^11^ ^a^	USA	2013	basal cell carcinoma	Caucasian	185/372	retrospective	hospital-based	leukocyte	quantitative PCR
Gabriella M. Anic *et al.*_SCC^11^ ^a^	USA	2013	squamous cell carcinoma	Caucasian	136/372	retrospective	hospital-based	leukocyte	quantitative PCR
Hongmei Nan *et al.*^12^	USA	2011	cutaneous melanoma	Caucasian	557/579	retrospective	population-based	leukocyte	quantitative PCR
Jiali Han *et al.*_melonoma^13^	USA	2009	melanoma	Caucasian	204/222	prospective	population-based	leukocyte	quantitative PCR
Jiali Han *et al.*_SCC^13^	USA	2009	squamous cell carcinoma	Caucasian	254/273	prospective	population-based	leukocyte	quantitative PCR
Jiali Han *et al.*_BCC^13^	USA	2009	basal cell carcinoma	Caucasian	282/306	prospective	population-based	leukocyte	quantitative PCR
Geyu Liang *et al.*_SCC^14^	USA	2011	squamous cell carcinoma	Caucasian	241/241	retrospective	population-based	leukocyte	quantitative PCR
Geyu Liang *et al.*_BCC^14^	USA	2011	basal cell carcinoma	Caucasian	623/1943	retrospective	population-based	leukocyte	quantitative PCR
Laura S. Burke *et al.*^15^	USA	2013	melanoma	Caucasian	119/208	retrospective	family-based	whole blood or EBV-transformed lymphocytes	quantitative PCR
Xifeng Wu *et al.*_RCC^16^	USA	2003	renal cell carcinoma	Caucasian	32/32	retrospective	population-based	leukocyte	Q-FISH
Xifeng Wu *et al.*_BLC^16^	USA	2003	bladder cancer	Caucasian	135/135	retrospective	population-based	leukocyte	Q-FISH
Lisa Mirabello *et al.*^17^	USA	2009	prostate cancer	Caucasian	612/1049	retrospective	population-based	leukocyte	quantitative PCR
B Julin *et al.*^18^	USA	2015	prostate cancer	Caucasian	922/935	retrospective	population-based	leukocyte	quantitative PCR
Lauren M. Hurwitz *et al.*^19^	USA	2014	prostate cancer	Caucasian	112/63	retrospective	family-based	leukocyte	quantitative PCR
Jonathan N. Hofmann *et al.*^20^	USA	2013	renal cell carcinoma	Caucasian	209/410	prospective	population-based	leukocyte	quantitative PCR
Jonathan N. Hofmann *et al.*_Caucasian^21^	USA	2011	renal cell carcinoma	Caucasian	658/550	retrospective	population-based	whole blood	quantitative PCR
Jonathan N. Hofmann *et al.*_African American^21^	USA	2011	renal cell carcinoma	African American	233/344	retrospective	population-based	whole blood	quantitative PCR
Monica McGrath *et al.*^22^	USA	2007	bladder cancer	Caucasian	184/192	retrospective	population-based	leukocyte	quantitative PCR
Karin Broberg *et al.*^23^	Sweden	2005	bladder cancer	Caucasian	63/93	retrospective	population-based	buccal cell	quantitative PCR
Andrew J. Pellatt *et al.*^24^	USA	2012	colon rectal cancer	Caucasian	525/746	retrospective	population-based	whole blood	quantitative PCR
Yong Cui *et al.*^25^	China	2012	colorectal cancer	Asian	512/549	retrospective	Population-based	leukocyte	quantitative PCR
Qin Qin *et al.*^26^	China	2014	colorectal cancer	Asian	628/1256	retrospective	hospital-based	leukocyte	quantitative PCR
Lifang Hou *et al.*^27^	USA	2009	gastric cancer	Caucasian	300/416	retrospective	population-based	leukocyte	quantitative PCR
Rosa Ana Risques *et al.*^28^	USA	2007	esophageal adenocarcinoma	Caucasian	38/300	prospective	population-based	leukocyte	quantitative PCR
Qianqian Yu *et al.*^29^	China	2014	esophageal squamous cell carcinoma	Asian	308/309	retrospective	hospital-based	lymphocyte	quantitative PCR
Jiangbo Du *et al.*^30^	China	2015	gastric cancer	Asian	1136/1102	retrospective	population-based	leukocyte	quantitative PCR
Xiaonan Liu *et al.*^31^	China	2009	gastric cancer	Asian	396/376	retrospective	hospital-based	leukocyte	quantitative PCR
Jinliang Xing *et al.*^32^	USA	2009	esophageal cancer	Caucasian	94/92	retrospective	hospital-based	leukocyte	quantitative PCR
Maria M. Gramatges *et al.*^33^	USA	2010	breast cancer	Caucasian	102/50	retrospective	population-based	leukocyte	quantitative PCR
Andrew J. Pellatt *et al.*^34^	USA	2013	breast cancer	Caucasian	728/720	retrospective	population-based	leukocyte	quantitative PCR
Jing Shen *et al.*^35^	USA	2009	breast cancer	Caucasian	1026/1070	retrospective	population-based	leukocyte	quantitative PCR
Immaculata *De Vivo et al.*^36^	USA	2009	breast cancer	Caucasian	896/917	prospective	population-based	leukocyte	quantitative PCR
Sangmi Kim *et al.*^37^	USA	2011	breast cancer	Caucasian	342/735	prospective	population-based	leukocyte	quantitative PCR
Jing Shen *et al.*^38^	USA	2007	breast cancer	Caucasian	283/347	retrospective	family-based	leukocyte	quantitative PCR
Yun-Ling Zheng *et al.*^39^	USA	2010	breast cancer	Caucasian	292/335	retrospective	population-based	leukocyte	Q-FISH
Shimian Qu *et al.*^40^	USA	2012	breast cancer	Asian	601/695	prospective	population-based	leukocyte	quantitative PCR
Xifeng Wu *et al.*_LC^16^	USA	2003	lung cancer	Caucasian	54/54	retrospective	population-based	leukocyte	Q-FISH
Min Shen *et al.*^41^	USA	2011	lung cancer	Caucasian	230/229	prospective	population-based	leukocyte	quantitative PCR
Qing Lan *et al.*^42^	USA	2013	lung cancer	Asian	215/215	prospective	population-based	leukocyte	quantitative PCR
Beatriz Sanchez-Espiridion *et al.*_LAC^43^	USA	2014	lung adenocarcinoma	Caucasian	706/706	retrospective	hospital-based	leukocyte	quantitative PCR
Beatriz Sanchez-Espiridion *et al.*_LSCC^43^	USA	2014	lung squamous cell carcinoma	Caucasian	320/320	retrospective	hospital-based	leukocyte	quantitative PCR
Wei Jie Seow *et al.*^44^	USA	2014	lung cancer	Caucasian	847/847	prospective	Population-based	leukocyte	quantitative PCR
Bing Sun *et al.*^45^	USA	2015	lung cancer	Caucasian	191/207	retrospective	Population-based hospital-based	lymphocyte	Q-FISH
Jin Sung Jang *et al.*^46^	Korea	2008	lung cancer	Asian	243/243	retrospective	hospital-based	leukocyte	quantitative PCR
Yang Zhang *et al.*_OCC^47^ ^a^	USA	2013	oral cavity cancer	Caucasian	137/335	retrospective	hospital-based	lymphocyte	quantitative PCR
Yang Zhang *et al.*_OPC^47^ ^a^	USA	2013	oropharyngeal squamous cell carcinoma	Caucasian	188/335	retrospective	hospital-based	lymphocyte	quantitative PCR
Da-Tian Bau *et al.*^48^	USA	2013	oral squamous cell carcinoma	Caucasian	92/394	retrospective	hospital-based	leukocyte	quantitative PCR
Xifeng Wu *et al.*_HNC^16^	USA	2003	head and neck cancer	Caucasian	92/92	retrospective	population-based	leukocyte	Q-FISH
Qing Lan *et al.*^49^	USA	2009	non-Hodgkin lymphoma	Caucasian	107/107	retrospective	population-based	leukocyte	quantitative PCR
Fatemeh Saberi Hosnijeh *et al.*^50^	Iran	2014	B-cell lymphoma	Caucasian	414/414	retrospective	population-based	leukocyte	quantitative PCR
Thomas A. Widmann *et al.*^51^	Germany	2007	non-Hodgkin’s lymphoma	Caucasian	40/40	retrospective	hospital-based	lymphocyte	Flow-FISH
Juan Liu *et al.*^52^	China	2011	hepatitis B virus-related hepatocellular carcinoma	Asian	240/240	retrospective	hospital-based	leukocyte	quantitative PCR
Shannon M. Lynch *et al.*^53^	USA	2013	pancreatic cancer	Caucasian	193/660	prospective	population-based	leukocyte	quantitative PCR
Xiaoying Fu *et al.*^54^	USA	2012	hepatocellular carcinoma	Asian	140/280	retrospective	hospital-based	circulating cell-free serum DNA	quantitative PCR
Daniele Campa *et al.*^55^	Germany	2014	myeloma	Caucasian	140/468	retrospective	population-based	leukocyte	quantitative PCR
Kathryn L. Terry *et al.*^56^	USA	2012	ovarian cancer	Caucasian	911/947	retrospective	population-based	leukocyte	quantitative PCR
Farzana Walcott *et al.*^57^	USA	2013	glioma	Caucasian	101/198	prospective	population-based	blood, buffy coat, or buccal cells	quantitative PCR
Jennifer Prescott *et al.*^58^	USA	2010	endometrial cancer	Caucasian	279/791	prospective	population-based	leukocyte	quantitative PCR
Maren Weischer *et al.*^59^	Denmark	2013	mixed	Caucasian	3142/41169	prospective	Population-based	leukocyte	quantitative PCR
Lisa Mirabello *et al.*^60^	USA	2009	ovarian cancer	Caucasian	99/100	retrospective	population-based	leukocyte	quantitative PCR
Peter Willeit *et al.*^61^	Italy	2010	mixed	Caucasian	92/695	prospective	population-based	leukocyte	quantitative PCR

^a^The controls were shared for different cancer types in the same publication.

**Table 2:** Summary of meta-analysis results for associations between telomere length and cancer risk.

Groups	Numbers		Heterogeneity		Associations (short vs. long)	
	Study	Case/Control	*P*	I^2^	OR(95% CI)	*P*
Overall	62	23379/68792	<0.001	0.90	1.10(0.98–1.23)	0.09
Populations						
Caucasian	51	18727/63183	<0.001	0.86	1.08(0.97–1.21)	0.18
Asian	10	4419/5265	<0.001	0.95	1.15(0.78–1.68)	0.49
African American	1	233/344	–	–	1.22(0.88–1.71)	0.23
Study design						
Prospective	16	7925/48662	<0.001	0.82	1.02(0.87–1.19)	0.80
Retrospective	46	15454/20130	<0.001	0.91	1.14(0.98–1.33)	0.10
Skin cancer						
Prospective	3	740/801	0.284	0.21	0.92(0.76–1.13)	0.44
Retrospective	7	2059/4087	<0.001	0.94	1.36(0.82–2.24)	0.23
Total	10	2799/4888	<0.001	0.91	1.17(0.83–1.66)	0.37
Tumors of urogenital system						
Prospective	1	209/410	–	–	0.92(0.66–1.28)	0.61
Retrospective	9	2951/3393	<0.001	0.70	1.01(0.81–1.25)	0.95
Total	10	3160/3803	0.002	0.66	0.99(0.82–1.20)	0.95
Gastrointestinal tumor						
Prospective	1	38/300	–	–	1.92(0.95–3.90)	0.07
Retrospective	8	3899/4846	<0.001	0.80	1.60(1.30–1.97)	8.20E-06
Total	9	3937/5146	<0.001	0.78	1.62(1.33–1.97)	2.03E-06
Breast cancer						
Prospective	3	1839/2347	0.370	0	1.13(0.99–1.28)	0.06
Retrospective	5	2431/2522	<0.001	0.87	0.82(0.58–1.17)	0.28
Total	8	4270/4869	<0.001	0.82	0.96(0.78– 1.19)	0.70
Lung cancer						
Prospective	3	1292/1291	0.330	0.10	0.78(0.67–0.91)	1.69E-03
Retrospective	5	1514/1530	<0.001	0.94	1.01(0.53–1.93)	0.97
Total	8	2806/2821	<0.001	0.90	0.91(0.63–1.31)	0.60
Head and neck cancer						
Prospective	–					
Retrospective	4	509/1156	0.019	0.70	1.86(1.23–2.82)	3.50E-03
Total	4	509/1156	0.019	0.70	1.86(1.23–2.82)	3.50E-03
Lymphoma						
Prospective	–					
Retrospective	3	561/561	<0.001	0.90	1.31(0.44–3.84)	0.63
Total	3	561/561	<0.001	0.90	1.31(0.44–3.84)	0.63
Other types of cancer						
Prospective	5	3807/43513	<0.001	0.87	1.22(0.85–1.75)	0.29
Retrospective	5	1530/2035	<0.001	0.95	0.69(0.33–1.45)	0.32
Total	10	5337/45548	<0.001	0.94	0.92(0.64–1.32)	0.65

**Table 3:** Dose-response relationship between telomere length and cancer risk by cancer type.

Cancer type	Numbers		Heterogeneity		Associations (short vs. medium vs. long)	
	Study	Case/Control	*P*	I^2^	OR(95% CI)	*P*
Overall	59	22674/67727	<0.001	0.91	1.09(1.01–1.19)	0.037
Skin cancer	10	2799/4888	<0.001	0.94	1.11(0.83–1.49)	0.496
Tumors of urogenital system	10	3160/3803	<0.001	0.79	1.15(0.97–1.37)	0.113
Gastrointestinal tumor	8	3558/4749	<0.001	0.88	1.29(1.08–1.54)	4.24E-03
Breast cancer	8	4270/4869	<0.001	0.83	0.96(0.83–1.11)	0.603
Lung cancer	8	2805/2823	<0.001	0.90	1.11(0.86–1.42)	0.415
Head and neck cancer	2	184/486	0.284	0.13	2.30(1.74–3.02)	2.85E-09
Lymphoma	3	561/561	<0.001	0.87	0.99(0.53–1.83)	0.970
