# Supplementary material for: The association between telomere length and cancer risk in population studies
Source: Sci Rep. 2016 Feb 26;6:22243. doi: 10.1038/srep22243 (PMC4768100; doi:10.1038/srep22243)
Supplement: Supplementary Information [file srep22243-s1.doc]

**The association between telomere length and cancer risk in population studies**

**SUPPLEMENTARY INFORMATION**

Xun Zhu^1†^, Wei Han^2†^, Wenjie Xue^1†^, Yuxia Zou^1^, Cuiwei Xie^1^, Jiangbo Du^1^, Guangfu Jin^1*^

^1^ Department of Epidemiology and Biostatistics, the Collaborative Innovation Center for Cancer Personalized Medicine, School of Public Health, Nanjing Medical University, Nanjing 211166, China

^2^ Xuzhou Center for Disease Control and Prevention, Xuzhou, 221003, China

^†^Zhu X, Han W and Xue W contributed equally to this work.

^*^**Correspondence to**: Guangfu Jin, Department of Epidemiology and Biostatistics, School of Public Health, Nanjing Medical University, Nanjing 211166, China, Tel: +86-25-8686-8437, Fax: +86-25-8686-8499, E-mail: guangfujin@njmu.edu.cn.

Table S1. Summary of extracted data from each study and respective association results

| Study [Reference] | Year | Sample size (case/control) | Short vs. Long | | |  | Short vs. Medium vs. Long | | |
| --- | --- | --- | --- | --- | --- | --- | --- | --- | --- |
|  |  |  | No. of case | No. of control | OR(95%CI) |  | No. of case | No. of control | OR(95%CI) |
| Gabriella M. Anic et al_melonoma [11] | 2013 | 198/372 | 91/107 | 247/125 | 0.43(0.30-0.61) |  | 123/44/18 | 122/125/125 | 2.70(2.08-3.50) |
| Gabriella M. Anic et al_BCC [11] ^a^ | 2013 | 185/372 | 167/18 | 247/125 | 4.70(2.76-7.99) |  | 19/72/107 | 122/125/125 | 0.47(0.37-0.60) |
| Gabriella M. Anic et al_SCC [11] ^a^ | 2013 | 136/372 | 129/7 | 247/125 | 9.33(4.23-20.56) |  | 97/32/7 | 122/125/125 | 3.50(2.53-4.84) |
| Hongmei Nan et al [12] | 2011 | 557/579 | 239/318 | 291/288 | 0.74(0.59-0.94) |  | 107/288/162 | 146/291/142 | 0.80(0.68-0.95) |
| Jiali Han et al_melonoma [13] | 2009 | 204/222 | 92/112 | 113/109 | 0.79(0.54-1.16) |  | 75/150/57 | 76/155/75 | 1.13(0.90-1.43) |
| Jiali Han et al_SCC [13] | 2009 | 254/273 | 115/139 | 136/137 | 0.83(0.59-1.17) |  | 48/93/63 | 55/112/55 | 0.87(0.66-1.13) |
| Jiali Han et al_BCC [13] | 2009 | 282/306 | 149/133 | 152/154 | 1.14(0.82-1.57) |  | 56/137/61 | 69/137/67 | 0.95(0.74-1.21) |
| Geyu Liang et al_BCC [14] | 2011 | 623/1943 | 300/323 | 972/971 | 0.93(0.77-1.11) |  | 66/112/63 | 59/122/60 | 1.03(0.81-1.32) |
| Geyu Liang et al_SCC [14] | 2011 | 241/241 | 123/118 | 122/119 | 1.02(0.71-1.45) |  | 156/305/162 | 482/974/487 | 0.99(0.87-1.12) |
| Laura S. Burke et al [15] | 2013 | 119/208 | 43/76 | 69/139 | 1.14(0.71-1.83) |  | 34/42/43 | 70/69/69 | 0.89(0.67-1.17) |
| Xifeng Wu et al_RCC [16] | 2003 | 32/32 | 10/22 | 16/16 | 0.45(0.16-1.26) |  | 15/16/1 | 8/16/8 | 2.86(1.25-6.55) |
| Xifeng Wu et al_BLC [16] | 2003 | 135/135 | 52/83 | 67/68 | 0.64(0.39-1.03) |  | 42/86/7 | 31/71/33 | 2.03(1.36-3.02) |
| Lisa Mirabello et al [17] | 2009 | 612/1049 | 291/321 | 543/506 | 0.84(0.69-1.03) |  | 142/326/144 | 292/504/253 | 0.92(0.80-1.06) |
| B Julin et al [18] | 2015 | 922/935 | 420/502 | 463/472 | 0.85(0.71-1.02) |  | 215/452/255 | 228/477/230 | 0.92(0.81-1.05) |
| Lauren M. Hurwitz et al [19] | 2014 | 112/63 | 54/58 | 32/31 | 0.90(0.49-1.67) |  | 38/37/37 | 16/31/16 | 1.01(0.68-1.51) |
| Jonathan N. Hofmann et al [20] | 2013 | 209/410 | 100/109 | 205/205 | 0.92(0.66-1.28) |  | 51/113/45 | 103/205/102 | 1.06(0.83-1.34) |
| Jonathan N. Hofmann et al_Caucasian [21] | 2011 | 658/550 | 346/312 | 297/253 | 0.94(0.75-1.19) |  | 161/318/179 | 160/275/115 | 0.81(0.69-0.95) |
| Jonathan N. Hofmann et al_African American [21] | 2011 | 233/344 | 114/119 | 151/193 | 1.22(0.88-1.71) |  | 52/101/80 | 64/173/107 | 1.01(0.80-1.27) |
| Monica McGrath et al [22] | 2007 | 184/192 | 119/65 | 93/99 | 1.95(1.26-2.87) |  | 68/77/39 | 46/97/49 | 1.39(1.05-1.84) |
| Karin Broberg et al [23] | 2005 | 63/93 | 43/20 | 47/46 | 2.10(1.08-4.11) |  | 32/23/8 | 23/47/23 | 2.15(1.33-3.47) |
| Andrew J. Pellatt et al [24] | 2012 | 525/746 | 332/193 | 456/290 | 1.09(0.87-1.38) |  | 193/80/252 | 290/151/305 | 0.89(0.79-1.01) |
| Yong Cui et al [25] | 2012 | 512/549 | 329/183 | 329/220 | 1.20(0.94-1.54) |  | 186/72/183 | 219/110/220 | 1.01(0.88-1.16) |
| Qin Qin et al [26] | 2014 | 628/1256 | 353/275 | 628/628 | 1.28(1.06-1.56) |  | 208/264/156 | 418/420/418 | 1.14(1.01-1.29) |
| Lifang Hou et al [27] | 2009 | 300/416 | 184/116 | 208/208 | 1.59(1.17-2.14) |  | 121/119/60 | 104/208/104 | 1.47(1.19-1.81) |
| Rosa Ana Risques et al [28] | 2007 | 38/300 | 25/13 | 150/150 | 1.92(0.95-3.90) |  | -- | -- | -- |
| Qianqian Yu et al [29] | 2014 | 308/309 | 181/127 | 128/181 | 2.02(1.46-2.78) |  | 15/19/4 | 75/150/75 | 1.83(1.10-3.02) |
| Jiangbo Du et al [30] | 2015 | 1136/1102 | 722/414 | 506/596 | 2.05(1.73-2.43) |  | 579/255/302 | 337/338/337 | 1.42(1.28-1.57) |
| Xiaonan Liu et al [31] | 2009 | 396/376 | 269/127 | 189/187 | 2.12(1.58-2.84) |  | 159/186/51 | 94/190/94 | 1.76(1.43-2.17) |
| Jinliang Xing et al [32] | 2009 | 94/92 | 66/28 | 46/46 | 2.36(1.29-4.30) |  | 45/28/21 | 30/32/30 | 1.48(1.03-2.12) |
| Maria M. Gramatges et al [33] | 2010 | 102/50 | 41/61 | 38/12 | 0.21(0.10-0.45) |  | 20/46/36 | 22/26/2 | 0.31(0.18-0.53) |
| Andrew J. Pellatt et al [34] | 2013 | 728/720 | 298/430 | 369/351 | 0.66(0.54-0.81) |  | 148/342/238 | 183/366/171 | 0.76(0.65-0.88) |
| Jing Shen et al [35] | 2009 | 1026/1070 | 513/513 | 536/534 | 1.00(0.84-1.18) |  | 287/487/252 | 284/517/269 | 1.04(0.92-1.17) |
| Immaculata De Vivo et al [36] | 2009 | 896/917 | 466/430 | 466/451 | 1.05(0.87-1.26) |  | 223/452/221 | 231/463/223 | 0.99(0.87-1.12) |
| Sangmi Kim et al [37] | 2011 | 342/735 | 177/165 | 365/370 | 1.09(0.84-1.41) |  | 81/169/92 | 182/366/187 | 0.95(0.79-1.14) |
| Jing Shen et al [38] | 2007 | 283/347 | 153/130 | 172/175 | 1.20(0.87-1.64) |  | 81/141/61 | 86/174/87 | 1.16(0.93-1.45) |
| Yun-Ling Zheng et al [39] | 2010 | 292/335 | 161/131 | 167/168 | 1.24(0.90-1.69) |  | 90/142/60 | 86/167/82 | 1.20(0.96-1.50) |
| Shimian Qu et al [40] | 2012 | 601/695 | 396/205 | 417/278 | 1.29(1.03-1.62) |  | 277/119/205 | 278/139/278 | 1.16(1.03-1.31) |
| Xifeng Wu et al [16] | 2003 | 54/54 | 11/43 | 27/27 | 0.26(0.11-0.60) |  | 30/22/2 | 13/28/13 | 3.44(1.79-6.61) |
| Min Shen et al [41] | 2011 | 230/229 | 91/139 | 115/114 | 0.65(0.45-0.94) |  | 43/120/66 | 58/114/58 | 0.81(0.62-1.06) |
| Qing Lan et al [42] | 2013 | 215/215 | 123/92 | 143/72 | 0.67(0.46-1.00) |  | 54/69/92 | 71/72/72 | 0.77(0.61-0.97) |
| Beatriz Sanchez-Espiridion et al_LAC [43] | 2014 | 706/706 | 299/407 | 364/342 | 0.69(0.56-0.85) |  | 140/338/228 | 176/357/173 | 0.77(0.67-0.90) |
| Beatriz Sanchez-Espiridion et al_LSCC [43] | 2014 | 320/320 | 195/125 | 159/161 | 1.58(1.15-2.16) |  | 115/141/64 | 82/159/79 | 1.33(1.07-1.66) |
| Wei Jie Seow et al [44] | 2014 | 847/847 | 394/453 | 429/418 | 0.85(0.70-1.03) |  | 193/400/254 | 214/423/210 | 0.86(0.75-0.98) |
| Bing Sun et al [45] | 2015 | 191/207 | 93/98 | 106/101 | 0.90(0.61-1.34) |  | 50/91/50 | 49/110/49 | 1.00(0.76-1.32) |
| Jin Sung Jang et al [46] | 2008 | 243/243 | 185/58 | 122/121 | 3.16(2.15-4.66) |  | 84/149/10 | 60/123/60 | 2.15(1.60-2.88) |
| Yang Zhang et al [47] | 2013 | 137/335 | 56/81 | 128/207 | 1.12(0.75-1.68) |  | -- | -- | -- |
| Yang Zhang et al [47] ^a^ | 2013 | 188/335 | 95/93 | 128/207 | 1.65(1.15-2.37) |  | -- | -- | -- |
| Da-Tian Bau et al [48] | 2013 | 92/394 | 67/25 | 197/197 | 2.68(1.63-4.42) |  | 42/40/10 | 99/197/98 | 2.05(1.46-2.90) |
| Xifeng Wu et al [16] | 2003 | 92/92 | 68/24 | 46/46 | 2.83(1.53-5.26) |  | 54/30/8 | 23/47/22 | 2.80(1.78-4.43) |
| Qing Lan et al [49] | 2009 | 107/107 | 34/73 | 53/54 | 0.47(0.27-0.83) |  | 16/44/47 | 27/53/27 | 0.57(0.39-0.83) |
| Fatemeh Saberi Hosnijeh et al [50] | 2014 | 414/414 | 187/227 | 210/204 | 0.80(0.61-1.05) |  | 83/197/134 | 103/207/104 | 0.79(0.65-0.95) |
| Thomas A. Widmann et al [51] | 2007 | 40/40 | 37/3 | 20/20 | 12.33(3.26-46.63) |  | 19/20/1 | 10/20/10 | 2.99(1.42-6.32) |
| Juan Liu et al [52] | 2011 | 240/240 | 28/212 | 120/120 | 0.13(0.08-0.21) |  | 7/76/157 | 60/120/60 | 0.23(0.16-0.31) |
| Shannon M. Lynch et al [53] | 2013 | 193/660 | 73/120 | 326/334 | 0.62(0.45-0.87) |  | 38/90/65 | 164/333/163 | 0.75(0.60-0.95) |
| Xiaoying Fu et al [54] | 2012 | 140/280 | 55/85 | 140/140 | 0.65(0.43-0.98) |  | 26/64/50 | 70/140/70 | 0.71(0.53-0.95) |
| Daniele Campa et al [55] | 2014 | 140/468 | 55/85 | 230/238 | 0.67(0.46-0.98) |  | 22/73/45 | 125/224/119 | 0.70(0.54-0.92) |
| Kathryn L. Terry et al [56] | 2012 | 911/947 | 594/317 | 619/328 | 0.99(0.82-1.20) |  | 317/275/319 | 328/305/314 | 0.98(0.87-1.09) |
| Farzana Walcott et al [57] | 2013 | 101/198 | 68/33 | 132/66 | 1.03(0.62-1.72) |  | 36/32/33 | 65/67/66 | 1.05(0.79-1.41) |
| Jennifer Prescott et al [58] | 2010 | 279/791 | 146/133 | 385/406 | 1.16(0.88-1.52) |  | 69/145/65 | 193/395/203 | 1.06(0.87-1.28) |
| Maren Weischer et al [59] | 2013 | 3142/41169 | 1773/1369 | 20184/20985 | 1.35(1.25-1.45) |  | 933/1557/652 | 9903/20734/10532 | 1.24(1.17-1.30) |
| Lisa Mirabello et al [60] | 2009 | 99/100 | 85/14 | 67/33 | 2.99(1.48-6.04) |  | 45/40/14 | 33/34/33 | 1.70(1.17-2.46) |
| Peter Willeit et al [61] | 2010 | 92/695 | 79/13 | 443/252 | 3.46(1.88-6.34) |  | 47/32/13 | 217/226/252 | 1.94(1.45-2.58) |

^a^ The controls were shared for different cancer types in the same publication.

Table S2 Summary of meta-analysis results for associations between telomere length and cancer risk among different ethnicity.

| Groups | Numbers | |  | Heterogeneity | |  | Associations(short vs. long) | |
| --- | --- | --- | --- | --- | --- | --- | --- | --- |
|  | Study | Case/Control |  | *P* | I^2^ |  | OR(95%CI) | *P* |
| Overall | 62 | 23379/68792 |  | <0.001 | 0.90 |  | 1.10(0.98-1.23) | 0.09 |
| Populations |  |  |  |  |  |  |  |  |
| Caucasian | 51 | 18727/63183 |  | <0.001 | 0.86 |  | 1.08(0.97-1.21) | 0.18 |
| Asian | 10 | 4419/5265 |  | <0.001 | 0.95 |  | 1.15(0.78-1.68) | 0.49 |
| African American | 1 | 233/344 |  | -- | -- |  | 1.22(0.88-1.71) | 0.23 |
| Skin cancer |  |  |  |  |  |  |  |  |
| Caucasian | 10 | 2799/4888 |  | <0.001 | 0.91 |  | 1.17(0.83-1.66) | 0.37 |
| Asian | -- | -- |  | -- | -- |  | -- | -- |
| Total | 10 | 2799/4888 |  | <0.001 | 0.91 |  | 1.17(0.83-1.66) | 0.37 |
| Tumors of urogenital system |  |  |  |  |  |  |  |  |
| Caucasian | 9 | 2927/3459 |  | 0.002 | 0.67 |  | 0.97(0.79-1.19) | 0.76 |
| African American | 1 | 233/344 |  | -- | -- |  | 1.22(0.88-1.71) | 0.23 |
| Total | 10 | 3160/3803 |  | 0.002 | 0.66 |  | 0.99(0.82-1.20) | 0.95 |
| Gastrointestinal tumor |  |  |  |  |  |  |  |  |
| Caucasian | 4 | 957/1554 |  | 0.040 | 0.65 |  | 1.54(1.10-2.16) | 0.01 |
| Asian | 5 | 2980/3952 |  | <0.001 | 0.83 |  | 1.67(1.30-2.15) | 6.89E-05 |
| Total | 9 | 3937/5146 |  | <0.001 | 0.78 |  | 1.62(1.33-1.97) | 2.03E-06 |
| Breast cancer |  |  |  |  |  |  |  |  |
| Caucasian | 7 | 3669/4174 |  | <0.001 | 0.82 |  | 0.91(0.73-1.15) | 0.45 |
| Asian | 1 | 601/695 |  | -- | -- |  | 1.29(1.03-1.62) | 0.03 |
| Total | 8 | 4270/4869 |  | <0.001 | 0.82 |  | 0.96(0.78-1.19) | 0.70 |
| Lung cancer |  |  |  |  |  |  |  |  |
| Caucasian | 6 | 2348/2363 |  | <0.001 | 0.82 |  | 0.80(0.59-1.08) | 0.15 |
| Asian | 2 | 458/458 |  | <0.001 | 0.97 |  | 1.46(0.32-6.65) | 0.62 |
| Total | 8 | 2806/2821 |  | <0.001 | 0.90 |  | 0.91(0.63-1.31) | 0.60 |
| Head and neck cancer |  |  |  |  |  |  |  |  |
| Caucasian | 4 | 509/1156 |  | 0.019 | 0.70 |  | 1.86(1.23-2.82) | 3.50E-03 |
| Asian | -- | -- |  | -- | -- |  | -- | -- |
| Total | 4 | 509/1156 |  | 0.019 | 0.70 |  | 1.86(1.23-2.82) | 3.50E-03 |
| Lymphoma |  |  |  |  |  |  |  |  |
| Caucasian | 3 | 561/561 |  | <0.001 | 0.90 |  | 1.31(0.44-3.84) | 0.63 |
| Asian | -- | -- |  | -- | -- |  | -- | -- |
| Total | 3 | 561/561 |  | <0.001 | 0.90 |  | 1.31(0.44-3.84) | 0.63 |
| Other types of cancer |  |  |  |  |  |  |  |  |
| Caucasian | 8 | 4975/49028 |  | <0.001 | 0.87 |  | 1.17(0.89-1.53) | 0.25 |
| Asian | 2 | 382/520 |  | <0.001 | 0.96 |  | 0.29(0.06-1.40) | 0.12 |
| Total | 10 | 5337/45548 |  | <0.001 | 0.94 |  | 0.92(0.64-1.32) | 0.65 |
